# Visual analysis of flow and diffusion of hemolytic agents and hematomas

**DOI:** 10.1186/s42492-020-00068-4

**Published:** 2021-01-30

**Authors:** Yafei Zhu, Mengyao Bao, Miaomiao Jiang, Jincai Chang, Jianzhong Cui

**Affiliations:** 1 College of Sciences, North China University of Science and Technology, Tangshan, 063210 Hebei China; 2 Department of Neurosurgery, Tangshan Gongren Hospital, Tangshan, 063000 Hebei China

**Keywords:** Flow diffusion, Navier-stokes equation, Tube flow, Fick’s law, Maxwell-Stefan diffusion equation, Visualization

## Abstract

The elimination of intracranial hematomas has received widespread attention and the interactions between hemolytic agents and hematomas have become a hot research topic. In this study, we used the Navier-Stokes equation to describe the flow control equation for hemolytic agents in a tube and used Fick’s law and the Maxwell-Stefan diffusion theory to describe the diffusion and mass transfer of hemolytic agents and hematomas. The physical fields and initial boundary conditions were set according to the parametric properties of the fluid and drainage tube. The COMSOL Multiphysics software was used to simulate the streamline distribution of hemolytic agents in a bifurcated drainage tube. Additionally, the diffusion behaviors of the hemolytic agents into hematomas were simulated and visual analysis of coupled multiphysics was performed to realize the digitization and visualization of engineering fluid problems and contribute to the field of medical engineering.

## Introduction

The entry of hemolytic agents into hematomas through drainage tubes is an essential component of the treatment of cerebral hemorrhages, but current medical software does not provide visual simulation functions for this process. Visualization would be of great significance for medical rehabilitation and drainage tube design. Therefore, it is necessary to simulate and analyze the transportation of hemolytic agents and the diffusion processes of hemolytic agents and hematomas visually.

Since the eighteenth century, theoretical fluid mechanics has gradually developed as a science in Europe. The theory of fluid mechanics has been significantly enriched and developed based on various experiments and the universal and accurate Navier-Stokes equations for fluid motion have been established [1, 2]. Computational fluid dynamics (CFD) have ushered in a period of rapid development [3]. The flow theory for single-component fluids is nearing perfection, which has inspired scholars to study the mass transfer of multiple components. The transport processes of multicomponent fluids involve collisions between the same and different molecules, and transmission mechanisms are relatively complicated [4], which means that this problem is not simply an extension of the single-component gas transmission process [5]. Therefore, the transport processes of multicomponent fluids should be considered as complex multi-field coupling problems [6]. For multicomponent liquids, there is no systematic and complete theory describing diffusion behavior. However, we can describe this behavior based on Fick’s law [7] and the diffusion model of the Maxwell-Stefan theory [8]. In classical physics, the phenomenon of self-diffusion is studied using an ideal gas as a model. The classic Fick’s law of self-diffusion was derived from such a model and this theoretical model has been analyzed based on the random thermal motion of microscopic particles [9]. However, it has been hypothesized that the value of Fick’s law of diffusion is also applicable to the transfer of dilute substances. In recent years, more accurate macroscopic models such as the Maxwell-Stefan model have received increasing attention [10]. Based on the rapid development of computer hardware technology, continuous improvement of calculation methods, and development of numerical analysis theories, a large number of domestic and foreign researchers have studied CFD and many numerical calculation methods suitable for various flow conditions have been developed [11]. These methods have laid a theoretical foundation for the numerical simulation of CFD.

Based on a background of CFD, this paper presents a study on the flow and diffusion systems of binary solutions under the effects of multiphysics fields. In this context, the molecules of different components tend to be evenly distributed in space, which reflects the migration of substances through space. We consider a basic method of mass transfer. According to Fick’s law and the Maxwell-Stefan theory, a fluid diffusion governing equation is derived and the CFD module in the COMSOL Multiphysics software is used to analyze the flow of hemolytic agents in a drainage tube visually, as well as the diffusion phenomenon that occurs when such agents enter high-concentration blood through a drainage tube. Model visualization analysis is the key to this research. It allows us to present abstract and difficult-to-observe diffusion phenomena in a visual form, yielding theoretical conditions for studying the diffusion behaviors of multicomponent liquids and even more complex liquids. This lays the foundation for the design of clinical drainage tubes and engineering equipment in other fields, including medical rehabilitation. The application of visualization technology to the medical and health industries will significantly improve the level of social medical care.

## Theory and methods

### Navier-stokes equation

The Navier-Stokes equation, as the most famous fluid-governing equation, is used to simulate various fluids found in nature. It is the most accurate and comprehensive mathematical model for describing fluids [12].

The commonly used incompressible non-viscous Navier-Stokes equation is defined as follows:

1 $$ \left\{\begin{array}{l}\frac{\partial u}{\partial t}+u\cdot \nabla u+\frac{1}{\rho}\nabla p=g\\ {}\nabla \cdot u=0\end{array}\right. $$

Here, *u* is the fluid velocity, *ρ* is the density, *p* is the pressure, and *g* is the gravitational acceleration.

The Navier-Stokes equation can reflect the basic mechanical laws of viscous fluid (also called real fluid) flow, so it has special significance for fluid mechanics [13]. It describes the incompressible equation for viscous fluids as follows:

2 $$ \left\{\begin{array}{l}\frac{\partial u}{\partial t}+u\cdot \nabla u+\frac{1}{\rho}\nabla p=g+\mu \nabla \cdot \nabla u\\ {}\nabla \cdot u=0\end{array}\right. $$

Here, *μ* is the coefficient of kinematic viscosity, which is used to measure fluid viscosity or is expressed as *υ*. ∇ symbol is a gradient operator, ∇⋅ is a divergence operator, and ∇ ⋅ ∇ is a Laplacian operator. The first equation is the momentum equation and the second equation is the mass equation, which defines the incompressibility of fluid velocity.

### Liquid flow in a tube

The flow of liquid in a tube is used as the basis for many fluid flow problems. As early as 1883, the British physicist Osborne Reynolds conducted a tube flow experiment using a circular tube and proposed a dimensionless combination parameter called the Reynolds number, which is represented by the symbol Re [14].

3 $$ \operatorname{Re}=\frac{\rho ud}{\mu }=\frac{ud}{v} $$

Here, *ρ* is the fluid density, *u* is the fluid flow rate, *d* is the tube diameter, *μ* is the dynamic viscosity, and *v* is the kinematic viscosity.

For tube flow with a circular cross-section, the critical Reynolds number of 2320 is used to define two flow regimes of laminar flow and turbulent flow. When the Reynolds number is Re ≤ 2320, the fluid is in laminar flow. When Re ≥ 4000, the fluid is in turbulent flow. The range between these numbers is called the critical zone.

The Mach number is a dimensionless quantity named after the Austrian physicist Mach (1836–1916) [15]. It is often simply referred to as the M number. The Mach number is the most important parameter for measuring the compressibility of fluid and it is defined as the ratio of the flow velocity to the speed of sound.

4 $$ Ma=\frac{u}{c} $$

Depending on the value of Mach number relative to a value of one, the fluid flow can be classified as a subsonic flow, supersonic flow, or transonic flow as follows:

$$ Ma\left\{\begin{array}{l}<1\kern0.75em \mathrm{Subsonic}\ \mathrm{flow}\\ {}\approx 1\kern0.75em \mathrm{Supersonic}\ \mathrm{flow}\\ {}>1\kern0.75em \mathrm{Transonic}\ \mathrm{flow}\end{array}\right. $$

### Law of diffusion

#### Fick’s law of diffusion

In 1855, Fick proposed a macroscopic law describing fluid diffusion called Fick’s law [16]. There are two main laws used to describe the steady-state diffusion and unsteady-state diffusion of fluids. Fick’s first law for describing steady-state diffusion is defined as follows. In a unit of time, the concentration gradient of fluid in a unit of cross-sectional area is proportional to the flow of the diffused material at that cross section, where the direction of the unit of cross-sectional area is perpendicular to the direction of diffusion. The flow of diffusing substances is also called the diffusion flux. The greater the concentration difference between units of the same fluid or different fluids, the greater the diffusion flux. This flux can be expressed by the following mathematical expression:

5 $$ {N}_i=-{D}_i\nabla {c}_i $$

For a substance (*i* = 1, 2), *N*_*i*_ is the molar flux (mol·m^−2^·s^−1^), *D*_*i*_ is the diffusion coefficient (m^2^/s), and *c*_*i*_ is the concentration (mol/m). The continuity equation is based on the conservation of mass.

6 $$ \frac{\partial {c}_i}{\partial t}+\nabla \cdot {N}_i=0 $$

Based on Fick’s first law, Fick’s second law can be derived to describe unsteady-state diffusion.

7 $$ \frac{\partial {c}_i}{\partial t}={D}_i{\nabla}^2{c}_i $$

The diffusion coefficient *D*_*i*_ can be set as a constant when describing the diffusion of chemical substances in water or other typical liquid solvents, such as dilute solutions.

According to the basic formula of Fick’s law, the one-dimensional mutual diffusion formula for binary components can be derived as follows:

8 $$ \frac{\partial {M}_1}{\partial t}=-{D}_{12}\frac{\partial {\rho}_1}{\partial x}\cdot A $$

In this formula, *D*_12_ is the inter-diffusion coefficient when component 1 and component 2 exhibit one-dimensional inter-diffusion, *M*_1_ is the mass number of transport component 1 over a time period Δ*t*, and *A* is the fitting parameter.

#### Maxwell-Stefan diffusion equation

The general form of the Maxwell-Stefan equation based on the theory of James Clerk Maxwell (1866) and Josef Stefan (1871) is written as follows:

9 $$ -{c}_i{\nabla}_T{\mu}_i= RT\sum \limits_{j=1}^n\frac{\xi_j{N}_i-{\xi}_i{N}_j}{{\underline{D}}_{ij}} $$

Here, *c*_*i*_ is the concentration of component *i*, *μ*_*i*_ is the chemical potential of *i*, *ξ*_*i*_ is the molar fraction of substance *i*, and *N*_*i*_ = *ξ*_*i*_*u*_*i*_ ∈ *R*_*d*_ is the molar flux of substance *i*. In the Maxwell-Stefan diffusion model, according to the friction coefficient between two components, the frictional force between component *i* and component *j* is calculated as $RT\frac{\xi_i\left({u}_i-{u}_j\right)}{{\underline{D}}_{ij}}$, where *R* is a constant, *T* represents the absolute temperature, and $\frac{RT}{{\underline{D}}_{ij}}$ can be regarded as a resistance coefficient. ${\underline{D}}_{ij}$ is the binary diffusion coefficient between the two substances. For physical reasons, the binary diffusion coefficient satisfies the symmetrical condition ${\underline{D}}_{ij}={\underline{D}}_{ji}$.

When an external field force exists, that external field force must be added to the driving force. Therefore, the driving force includes both external field forces and the chemical potential gradient [17]. The general form of the Maxwell-Stefan equation under an external field is written as follows:

10 $$ -{c}_i{\nabla}_T{\mu}_i+{c}_i\overrightarrow{f_i}= RT\sum \limits_{j=1}^n\frac{\xi_j{N}_i-{\xi}_i{N}_j}{{\underline{D}}_{ij}} $$

The expression −*c*_*i*_∇_*T*_*μ*_*i*_ in this equation represents the force acting on component *i* of the mixture per unit volume. ${c}_i\overrightarrow{f_i}$ represents the external field force acting on component *i* in the mixture per unit volume. The expressions to the left of the equal sign represent the total diffusion driving force acting on the component *i*.

## Algorithm flow

In real-world engineering problems, fluid flow and diffusion are very common phenomena. In this study, we used the COMSOL Multiphysics software to analyze these fluid phenomena visually and apply it to the medical and clinical fields. Simulation in COSMOL Multiphysics requires the following processes: establishing and selecting spatial dimensions, adding physical quantities, defining geometric models and physical parameters, dividing finite element meshes, solving, and visualizing via post-processing [18]. The general algorithm flow for COMSOL Multiphysics software simulation is outlined below:

Step 1: Select the required simulation model, list the required control equations, and define known parameters and necessary boundary conditions.

Step 2: Select the appropriate model dimensions to interface with physics elements.

Step 3: Select the required research status.

Step 4: Set the size of the working space according to the size of the simulation model and construct a geometric model.

Step 5: Set the boundary conditions and various physical parameters.

Step 6: Grid division (select the appropriate grid size for division).

Step 7: Derive and set appropriate solution conditions.

Step 8: Post-processing: Use the physical quantities outputted by the simulation to calculate the required physical quantities.

## Determination of initial boundary conditions

### Problem description

**Fig. 1:**
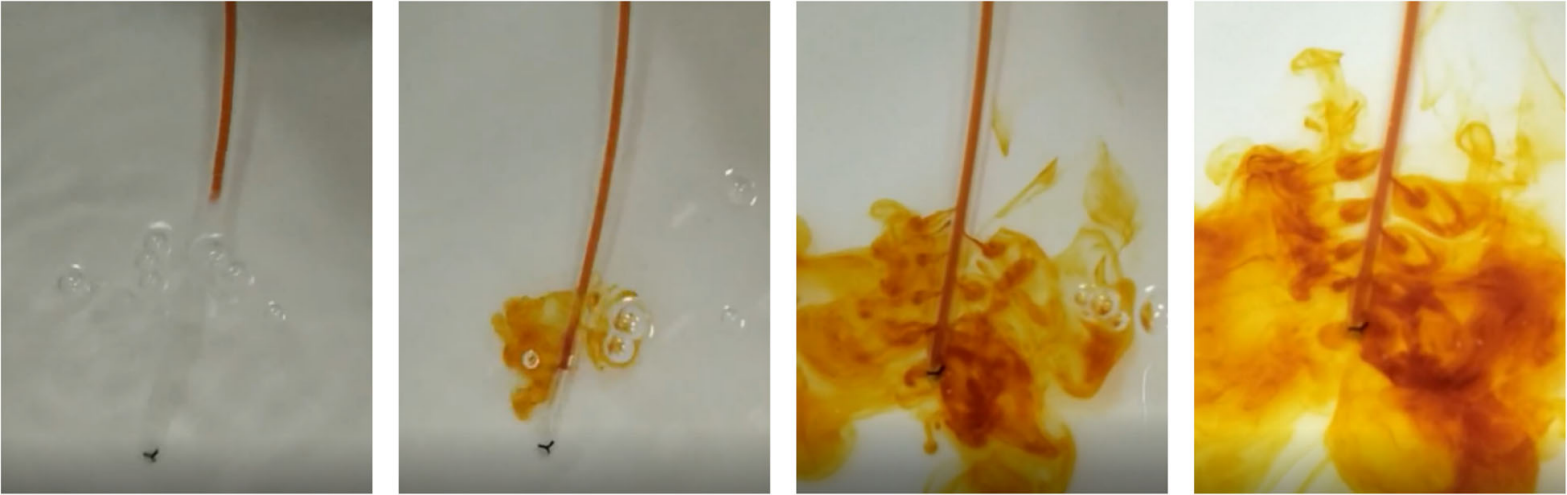
Physical experiments on binary liquid diffusion

This section mainly focuses on using the COMSOL Multiphysics software to simulate the flow of hemolytic agents and the convective diffusion between hemolytic agents and hematomas. The flow type is mainly the flow of a hemolytic agent in a tube and the type of convection diffusion is a mass transfer model that ignores heat transfer and chemical reactions. We divide the visualization process into two stages. The first stage focuses on the flow of a hemolytic agent through a drainage tube before it interacts with a hematoma. The second stage is the convective diffusion mass transfer that occurs when the two liquids come into contact. Two types of color differences are selected. Water and iodophor are used in physical experiments to embody the simulation process. These experiments are illustrated in the Fig. 1.

In this physical experiment, the iodophor was drained into the water tank at a certain initial speed through a drainage tube with a number of small holes. Each of the small holes had the same size and the phenomenon of diffusion into water occurred, as shown in the Fig. 1. It is clear that the liquid flow through the small drainage holes is not consistent and is distributed in a tower shape. To make the iodophor flow from each small hole uniformly, the diameter, angle, length, and other parameters of the drainage tube must be optimized.

### Geometric model

**Fig. 2:**
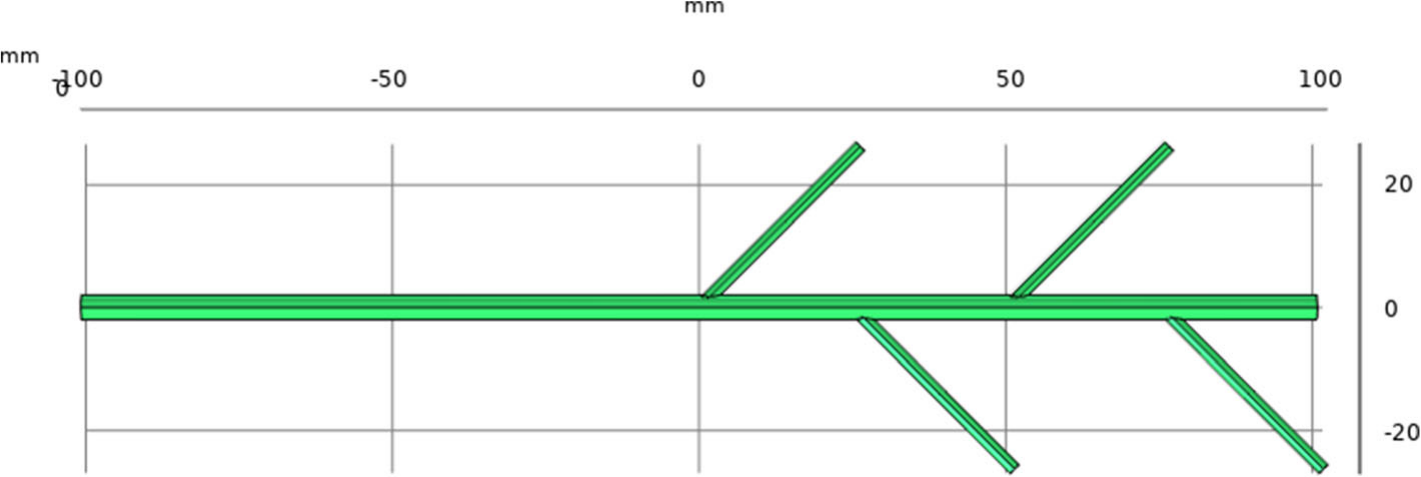
Bifurcated drainage tube design drawing

According to the background of the target problem and clinical needs, the type of tube in this study was set to a bifurcated circular tube to accelerate convective diffusion and mass transfer. To lay the foundation for further research and design a more efficient bifurcated drainage tube instrument, we initially designed an original bifurcated tube [19]. The relevant parameters were set such that the length of the main tube in the middle was 200 mm, the radius was 2 mm, the branch tubes were set at the center of the main tube, and one branch tube was installed every 25 mm. The branch angle of each branch tube is 45°, the length is 35.4 mm, the radius is 1 mm, the left end of the main tube is the inlet, and the right end of the main tube and the ends of the branch tubes are the outlets. The design of the bifurcated drainage tube is presented in the Fig. 2.

### Fluid properties

The two fluids simulated in this study were hemolytic agents and hematomas. The parameters of these fluids were set as shown in the Table 1.

**Table 1:** Hemolytic agent and hematoma parameter table

Fluid	Density (kg/m^3^)	Dynamic viscosity (Pa·s)
Hemolytic agent (urokinase)	30	1 × 10^−3^
Hematoma (high-concentration blood)	1340	5 × 10^−3^

Urokinase lysis is an exogenous plasminogen that can effectively decompose fibrin deposits and prevent blood clotting [20]. The chemical reactions involved in this process are relatively complicated. We ignored these chemical reactions when designing our physics interface and only performed flow and diffusion simulations.

Because there is no single method for the preparation of hemolytic agents, the required urokinase content differs for different patients, locations, and hematoma concentrations. Therefore, the densities and dynamic viscosities of prepared solutions also differ. The parameters of the hemolytic agent used in our simulations are based on the optimal dosage of 30 IU/mL of urokinase in sterile saline for the hemolytic agent for a brain hematoma [21]. The density of the urokinase solution is 30 kg/m^3^ according to unit conversion. To reflect diffusion behaviors accurately, we consider a hematoma as a fluid with a higher concentration. According to the physiological characteristics of blood, the dynamic viscosity of normal blood is three to four times that of a hemolytic agent prepared with saline. The viscosity of a hematoma is even higher. Here, the dynamic viscosity of a hematoma was set to 5 × 10^−3^ Pa·s.

### Boundary conditions

In the study presented in ref. [22], the initial boundary conditions for a water treatment plant system were determined. The initial boundary conditions for our study were derived in a similar manner and a CFD module was selected for simulation. By combining the flows of the hemolytic agent in the bifurcated tube and the diffusion reaction that occurs when the agent enters the hematoma (i.e., when the two physical fields of tube flow and material transfer are coupled) along the inner surface of the tube, the equation of motion satisfying no-slip tube wall conditions is *u*|_*r*_ = 0, where the velocity is *μ*_0_ = 0.05 m/s. The transfer mechanisms satisfied by the diffusion model are Fick’s law and the Maxwell-Stefan model. The additional mechanism is convection. The concentration of the hemolytic agent was set to *c*_0_ = 1 mol/m^3^ and the concentration of the hematoma was set to *c*_1_ = 3 mol/m^3^. The diffusion coefficient between the two fluids was set to *D* = 6.9 × 10^−11^ m^2^/s. Based on the particularity of the physical form of hematoma, it must be combined with a porous media dilute substance transfer module for simulation analysis. The porosity was set to *ε*_*p*_ = 0.21.

## Results and discussion

### Macroscopic visual analysis

As a hemolytic agent flows through a tube, there is loss along the way with a constant flow cross section. Additionally, where the flow cross section changes, there is a local loss of flow at bifurcations in the tube. Therefore, the pressure and velocity fields at different positions along the tube differ. Regarding tube properties, the initial boundary conditions are controlled by the following equations:

11 $$ A=\frac{\pi }{4}{d}_i^2 $$

12 $$ Z=\pi {d}_i $$

13 $$ {d}_h={d}_i $$

14 $$ \operatorname{Re}=\frac{\rho {ud}_h}{\mu } $$

Here, *d*_*i*_ is the tube outer wall diameter and *d*_*h*_ is the tube hydraulic diameter. It is assumed that these two values are equal, meaning the tube wall thickness is ignored. *A* is the tube cross-sectional area and *Z* is the tube wet circumference, meaning the circumference of the fluid flowing through the tube. According to the geometrical model parameters, the fluid property parameters and initial boundary value conditions given in first part of the main tube are *d*_*i*_ = *d*_*h*_ = 2 mm. The dynamic viscosity of the hemolytic agent is approximately *μ* = 1 × 10^−3^ Pa·s and the density is approximately ρ = 30 kg/m^3^. This information is entered into the expressions above to obtain a Reynolds number expression, denoted as Re. From this expression, we can calculate the Darcy friction factor, denoted as *f*_*D*_.

15 $$ {f}_D=8{\left[{\left(\frac{8}{\operatorname{Re}}\right)}^{12}+{\left({c}_A+{c}_B\right)}^{-1.5}\right]}^{\frac{1}{12}} $$

Here, *c*_*A*_ and *c*_*B*_ are the liquid concentrations at any two points A and B, respectively, which are expressed as follows:

$$ {c}_A={\left\{-2.57\ln \left[{\left(\frac{7}{\operatorname{Re}}\right)}^{0.9}+0.27\left(\frac{e}{d_h}\right)\right]\right\}}^{16} $$

$$ {c}_B={\left(\frac{37530}{\operatorname{Re}}\right)}^{16} $$

Here, *e* is the surface roughness (*e* = 0.0015 mm).

The fluid properties are defined by the following governing equations:

16 $$ \rho u\nabla \cdot \left({ue}_t\right)=-{\nabla}_tp\cdot {e}_t-\frac{1}{2}{f}_D\frac{\rho }{d_h}\left|u\right|u+F\cdot {e}_t $$

17 $$ {\nabla}_t\cdot \left( A\rho {ue}_t\right)=0 $$

After the flow trajectory of the fluid has been determined, the properties of the fluid are only related to time, where *F* is the volume force and *e*_*t*_ is the tangential vector of roughness over time.

The treatment of nondestructive tube joints is handled based on the following formula:

18 $$ {p}_{junction}={p}_i+\frac{1}{2}{\rho}_i{u}_i^2 $$

Here, *p*_*i*_ is the pressure of fluid *i*, *ρ*_*i*_ is the density of fluid *i*, and *u*_*i*_ is the velocity of fluid *i* flowing into a joint.

Based on the calculations above, we can obtain the three-dimensional pressure and velocity distribution results for a fluid flowing through a bifurcated tube with a height expression.

**Fig. 3:**
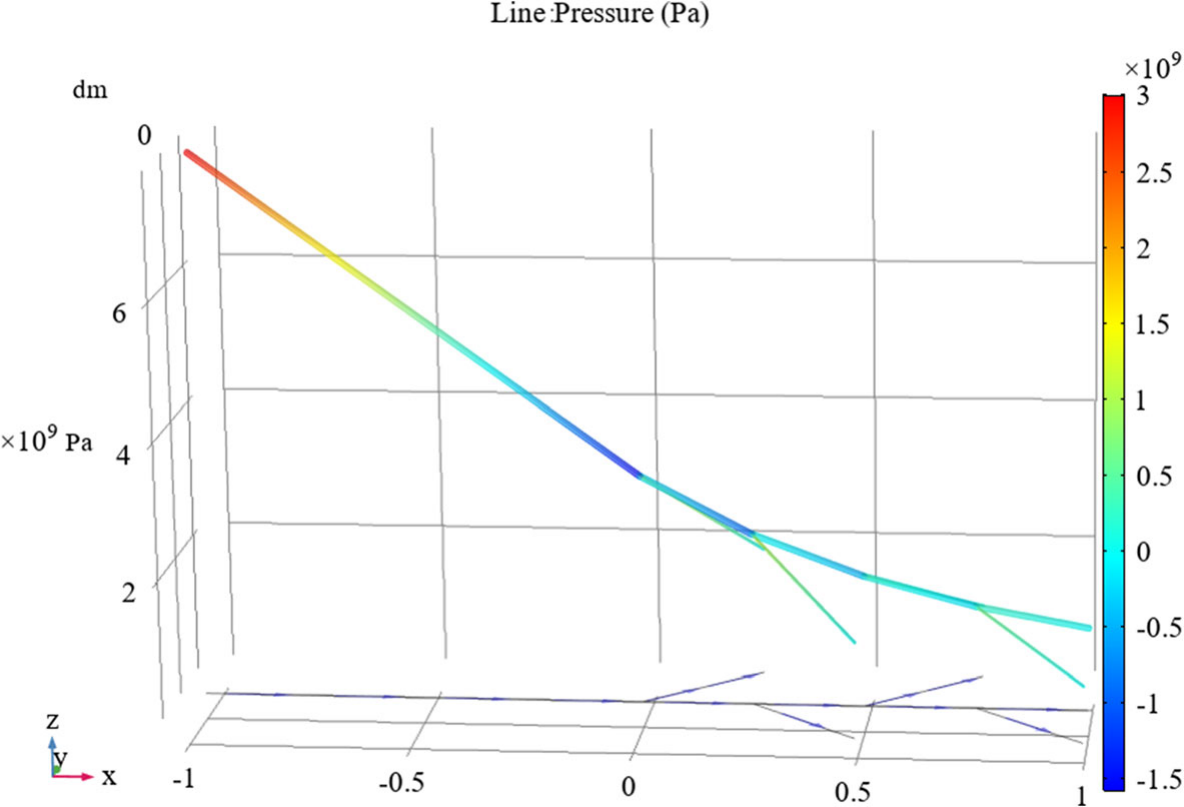
Relationship between tube pressure and position in the tube system

**Fig. 4:**
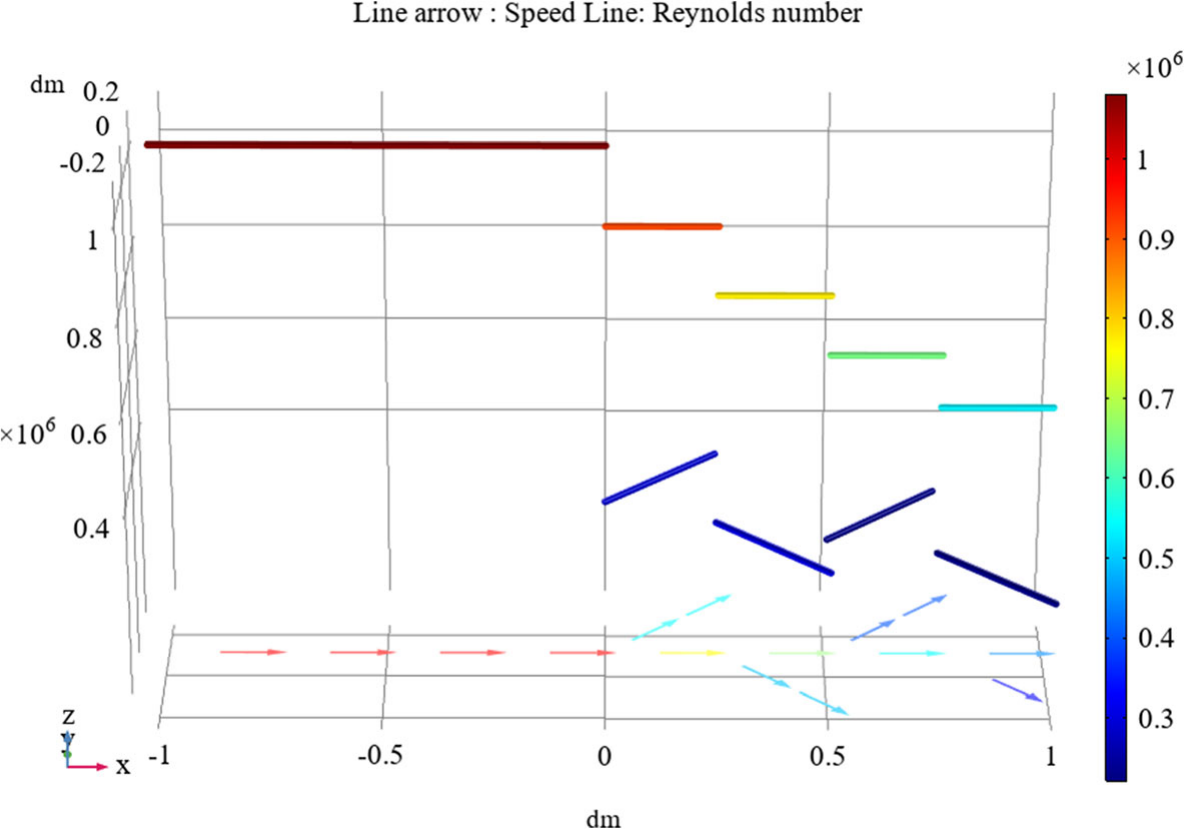
Velocity distribution

Figures 3 and 4 indicate that for a particular initial velocity and flow driving force, the initial pressure value is determined in the tube inlet stage and the fluid velocity change and pressure change through the entire tube network are virtually the same. Additionally, the hemolytic agent has a specific flow rate. With the extension of the tube and loss of energy consumption, the pressure gradually decreases and eventually trends toward zero. When the tube diameter is halved, accompanied by a local loss of energy consumption, the flow rate decreases. Because the fluid has an initial velocity, the flow velocity at the end of the nozzle will not drop to zero, but the fluid will slowly flow out of the drainage tube.

### Visual analysis of microstreamlines

For a given velocity and pressure in a bifurcated tube, the flow state is determined by the Reynolds number and Mach number of the liquid flowing through the tube. The parameters of the hemolytic agent and tube were fed into Eqs. (3) and (4) to calculate that the Reynolds numbers of the main tube and branch tubes are Re_1_ ≈ 0.3 < 2320 and Re_2_ ≈ 0.15 < 2320, respectively, while the Mach number of the fluid is *Ma* = 0.05 < 1. It can be determined that the flow of the hemolytic agent in both the main tube and branch tubes is laminar. The fluid flows at subsonic speeds, so its compressibility can be ignored. Therefore, the incompressible Navier-Stokes equation can be used to describe the flow of the hemolytic agent in the tube.

**Fig. 5:**
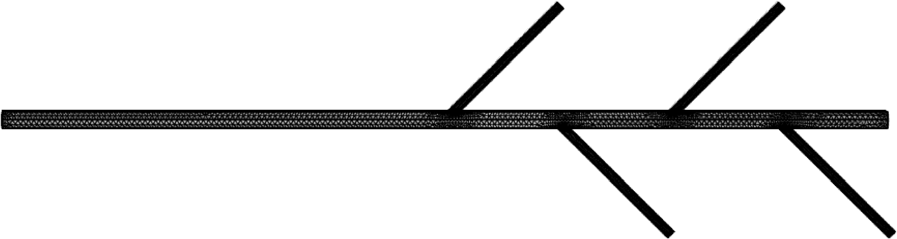
Mesh division of the bifurcated tube

The COMSOL Multiphysics software was used to generate a coarser mesh controlled by the physics field acting on a bifurcated tube, as shown in the Fig. 5.

**Fig. 6:**
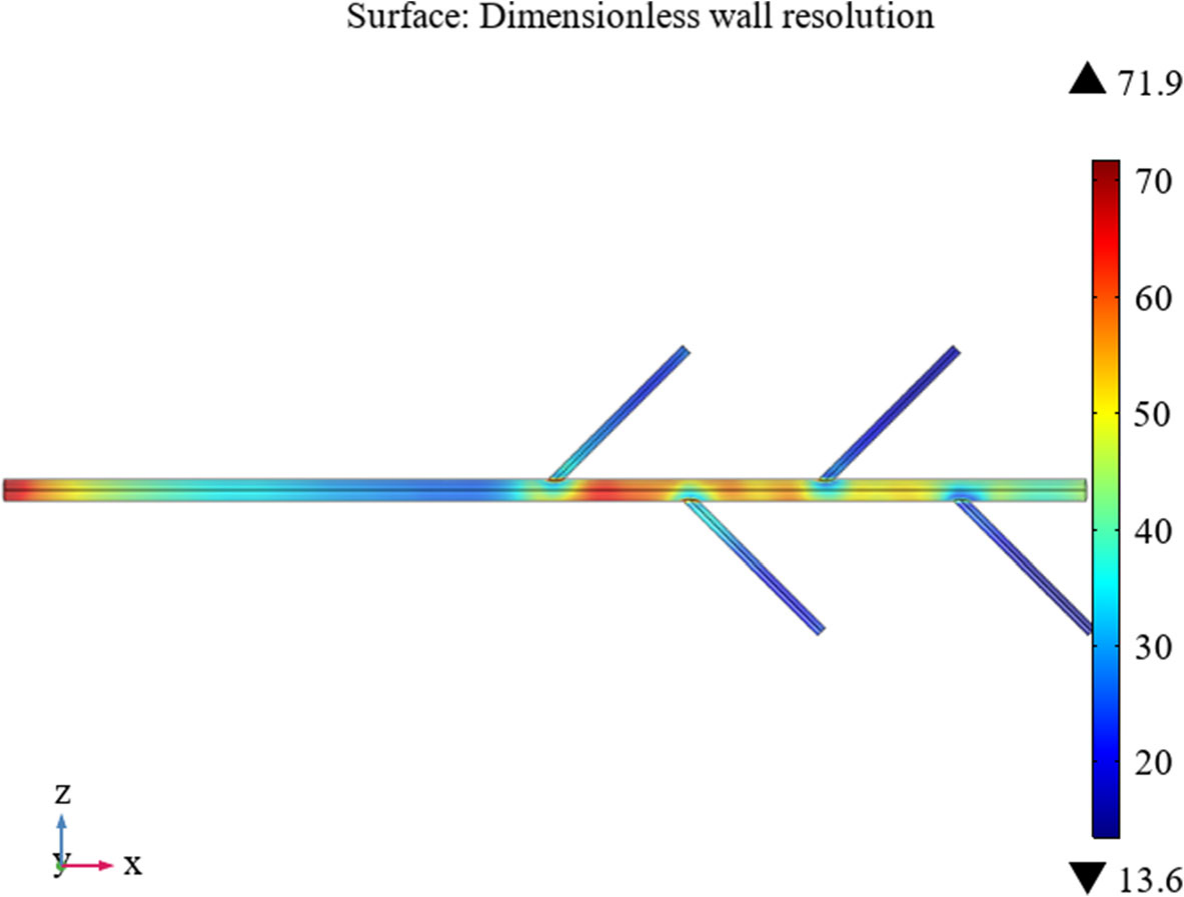
Wall resolution

The initial boundary conditions ensure that the motion equation satisfies the non-slip tube wall condition of *u*|_*r*_ = 0 and that the wall resolution of the bifurcated drainage tube can be obtained, as shown in the Fig. 6.

Figure 6 reveals that the wall lift force everywhere in the bifurcated tube is much lower than 100 viscous units, meaning the flow can be considered to be well resolved on the wall.

The fluid properties governing the equation of laminar flow are defined as follows:

19 $$ \backslash \mathrm{rho}\backslash \mathrm{left}\left(\mathrm{u}\backslash \mathrm{cdot}\backslash \mathrm{nabla}\backslash \mathrm{right}\right)\mathrm{u}=\backslash \mathrm{nabla}\backslash \mathrm{cdot}\backslash \mathrm{left}\Big(-\mathrm{pI}+\mathrm{K}\backslash \mathrm{right}+\mathrm{F} $$

20 $$ \rho \nabla \cdot u=0 $$

Here, *K* is the viscous stress, which is expressed by the following formula:

$$ K=\mu \left[\nabla u+{\left(\nabla u\right)}^T\right] $$

∇ ⋅(−*pI* + *K*) is the diffusion term of the governing equation and *F* is the volume force, which is the source term for the governing equation. Equation (20) satisfies the incompressible condition of fluid flow.

The inlet and outlet stages of the tube are set to be fully developed flows, where the inlet stage is constrained by a velocity field with an average velocity of *u*_*av*_ = 0.05 m/s and the outlet is constrained by the average pressure. Therefore, the governing equation can be written as follows:

21 $$ {\displaystyle \begin{array}{l}u\cdot t=0\\ {}\left(- pI+K\right)n=-{p}_{grad}n\end{array}} $$

**Fig. 7:**
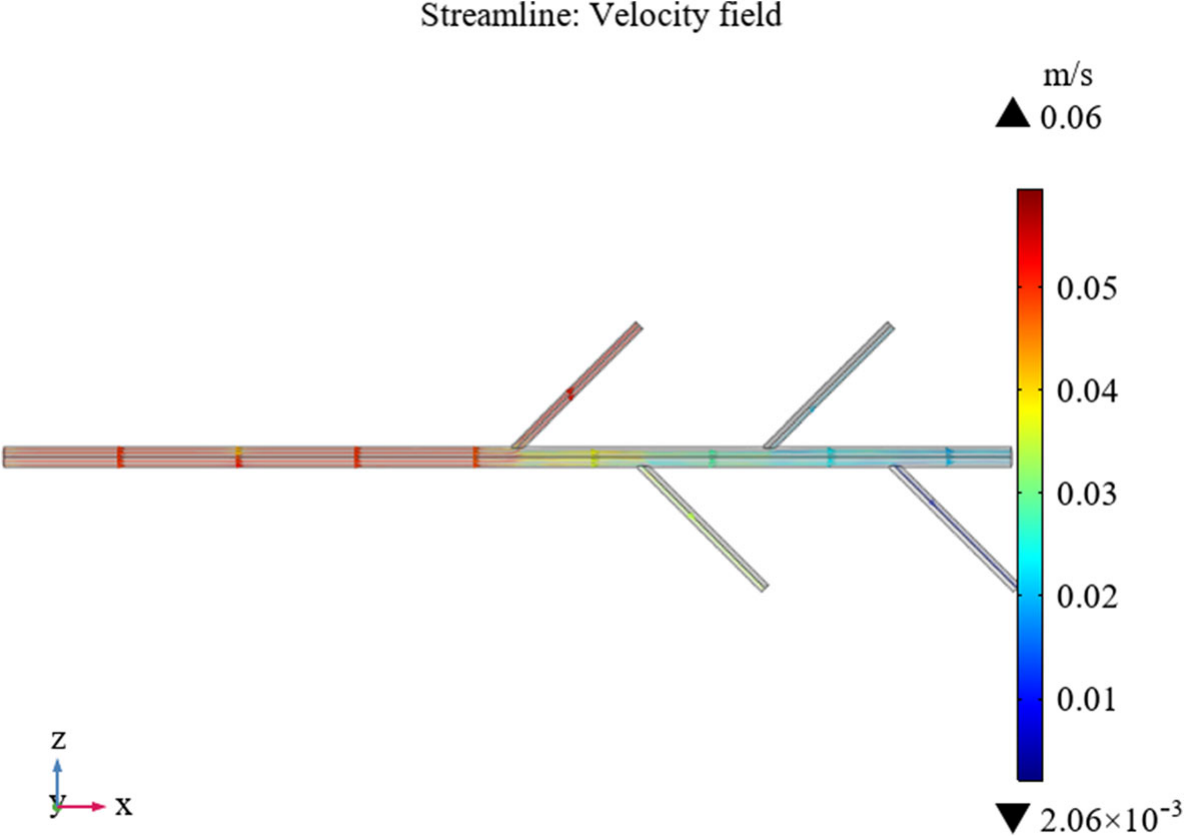
Velocity field streamline diagram

By combining this equation with the streamline visualization method [23], a three-dimensional streamline diagram of the liquid flow in the tube can be obtained (Fig. 7).

To facilitate observation, we enlarged the streamline diagram of the bifurcated drainage tube to obtain the following partially enlarged views.

**Fig. 8:**
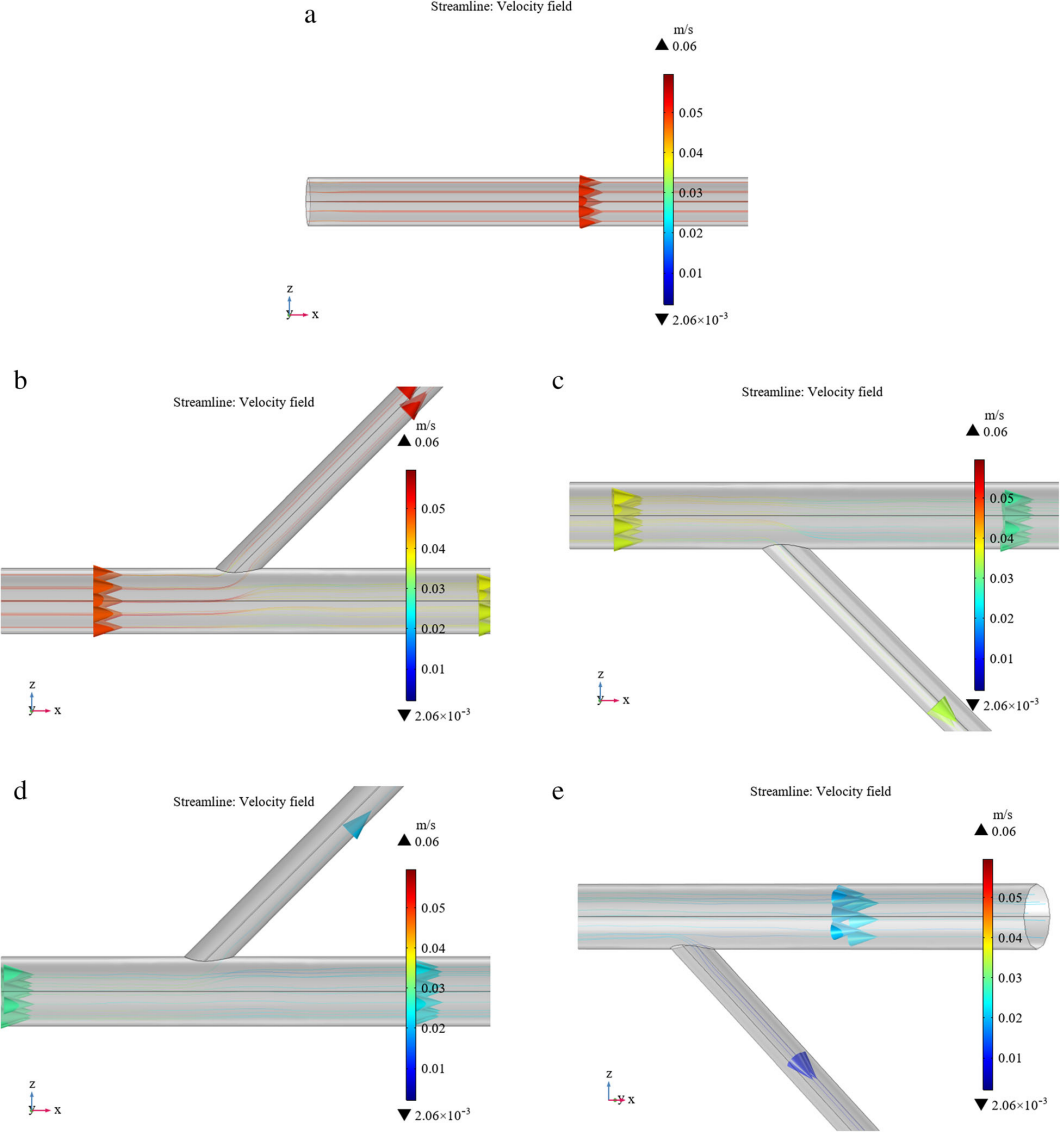
Local streamline diagram of the bifurcated tube velocity field. **a** Entry stage; **b** Part of the first bifurcation; **c** Part of the second bifurcation; **d** Part of the third bifurcation; **e** Part of the fourth bifurcation

Figure 8 presents a colorized streamline diagram of the local velocity amplitudes in the tube. The flow at the inlet stage is laminar, so the flow lines are distributed in parallel. In addition to the laminar flow at the root of each bifurcated tube, there are also turbulent flows, which are similar to the critical state of flow. As the fluid flows, the farther it travels from the inlet, the lower the fluid velocity.

### Hemolytic agent and hematoma diffusion simulation

The shape model of a hematoma was extracted from a computed tomography image of a patient with an intracranial hematoma and imported into the COMSOL Multiphysics software. The bifurcated drainage tube model was then imported and grid refinement of the drainage tube was performed as it acted on the hematoma, as shown in the figure below.

**Fig. 9:**
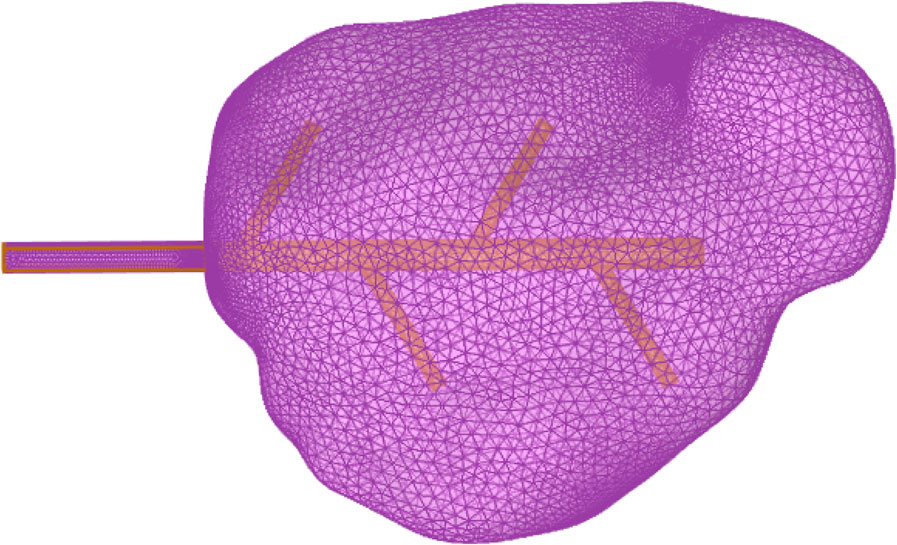
Meshing of the drainage tube and hematoma

The refined grid structure shown in Fig. 9 can be controlled by users. The cell size is calibrated to match the target fluid dynamics. Under conventional predefined conditions, the cell grid parameter size is divided and the maximum cell size is 2.01 mm, the minimum cell size is 0.601 mm, the maximum cell growth rate is 1.15, the curvature factor is 0.6, and the narrow-area resolution is 0.7. The element size scaling factor for refining angles is 0.35 and smoothing is performed across all removed control entities. The number of iterations is four and the maximum element depth to be processed is four units. Analysis was performed on this refined mesh using calculations and a preliminary interaction diagram of the hemolytic agent and hematoma was obtained. The concentration difference between the hemolytic agent and hematoma is defined by the concentration of the initial boundary conditions so that the system produces a diffusion phenomenon driven by concentration differences.

For the fluid flow in the free-flow region, the steady-state Navier-Stokes equation can be applied, where the law of mutual diffusion is derived from Fick’s law.

22 $$ \frac{\partial {M}_1}{\partial t}=-{D}_{12}\frac{\partial {\rho}_1}{\partial x}\cdot A $$

In this section, the Maxwell-Stefan diffusion model is incorporated [24], convection and porous media mass transfer mechanisms are added, and the mass conservation equation is adopted to obtain a convection-diffusion continuity equation under the transfer of dilute species.

23 $$ {\displaystyle \begin{array}{l}\frac{\partial \left({\varepsilon}_p{c}_i\right)}{\partial t}+\frac{\partial \left(\rho {c}_{p,j}\right)}{\partial t}+\nabla \cdot {N}_i+u\cdot \nabla {c}_i={R}_i+{S}_i,\\ {}\kern5.5em {N}_i=-{D}_{e,i}\nabla {c}_i.\end{array}} $$

Here, *ε*_*p*_ is the porosity (*ε*_*p*_ = 0.21 was derived from the calculation process), *R*_*i*_ is the total diffusion rate of substance *i*, and *S*_i_ is the source term. The flux expression has the form of Fick’s law.

The fluid diffusion coefficient is an isotropic coefficient.

24 $$ {D}_{e,i}=\frac{\varepsilon_p}{\tau_{F,i}}{D}_{F,i} $$

Here, *τ*_*F*, *i*_ is the effective diffusion coefficient model of the Millington-Quirk model.

$$ {\tau}_{F,i}={\varepsilon}_p^{-\frac{1}{3}} $$

**Fig. 10:**
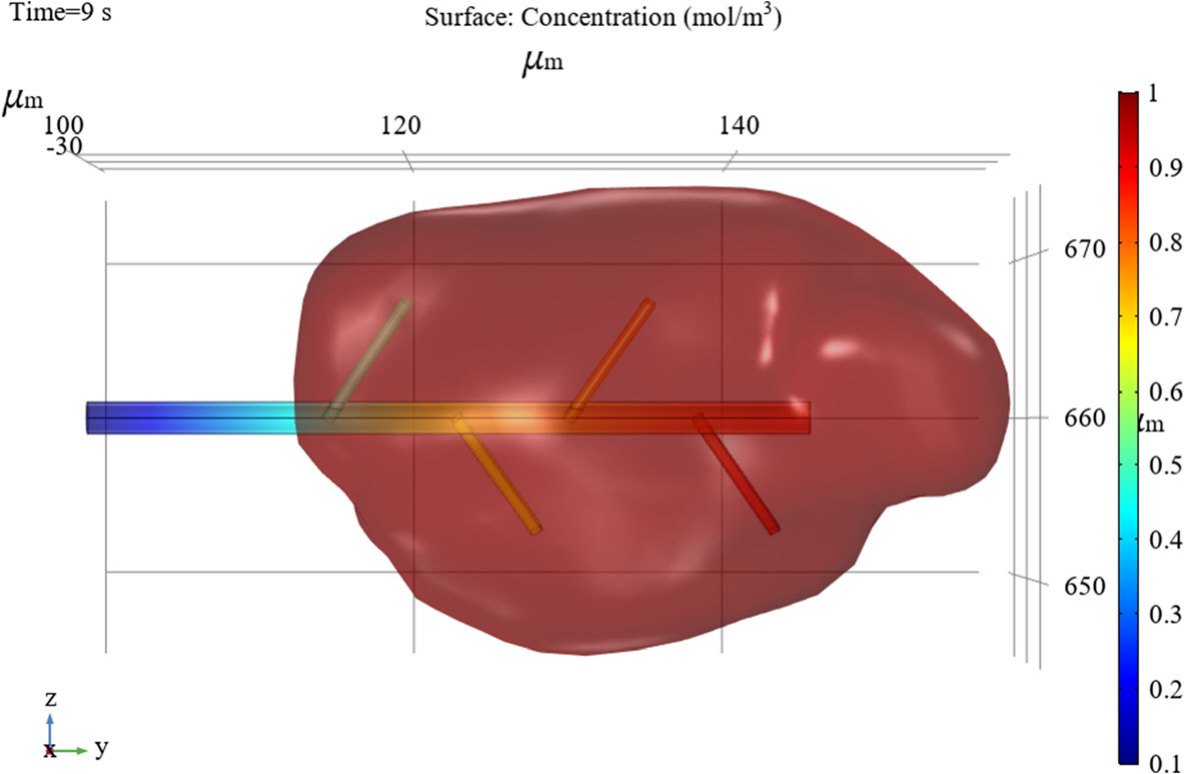
Concentrations on the wall of the bifurcated tube when the hemolytic agent flows

Figure 10 illustrates the concentration of the hemolytic agent on the wall surface of the tube when it drains into the hematoma over 9 s.

To obtain the diffusion concentration changes of the hemolytic agent and hematoma, the simulation target is placed in the interactive area between the hematoma and bifurcated drainage tube, and post-processing is performed on the COMSOL results to account for the concentration surface two-dimensional drawing group animation.

**Fig. 11:**
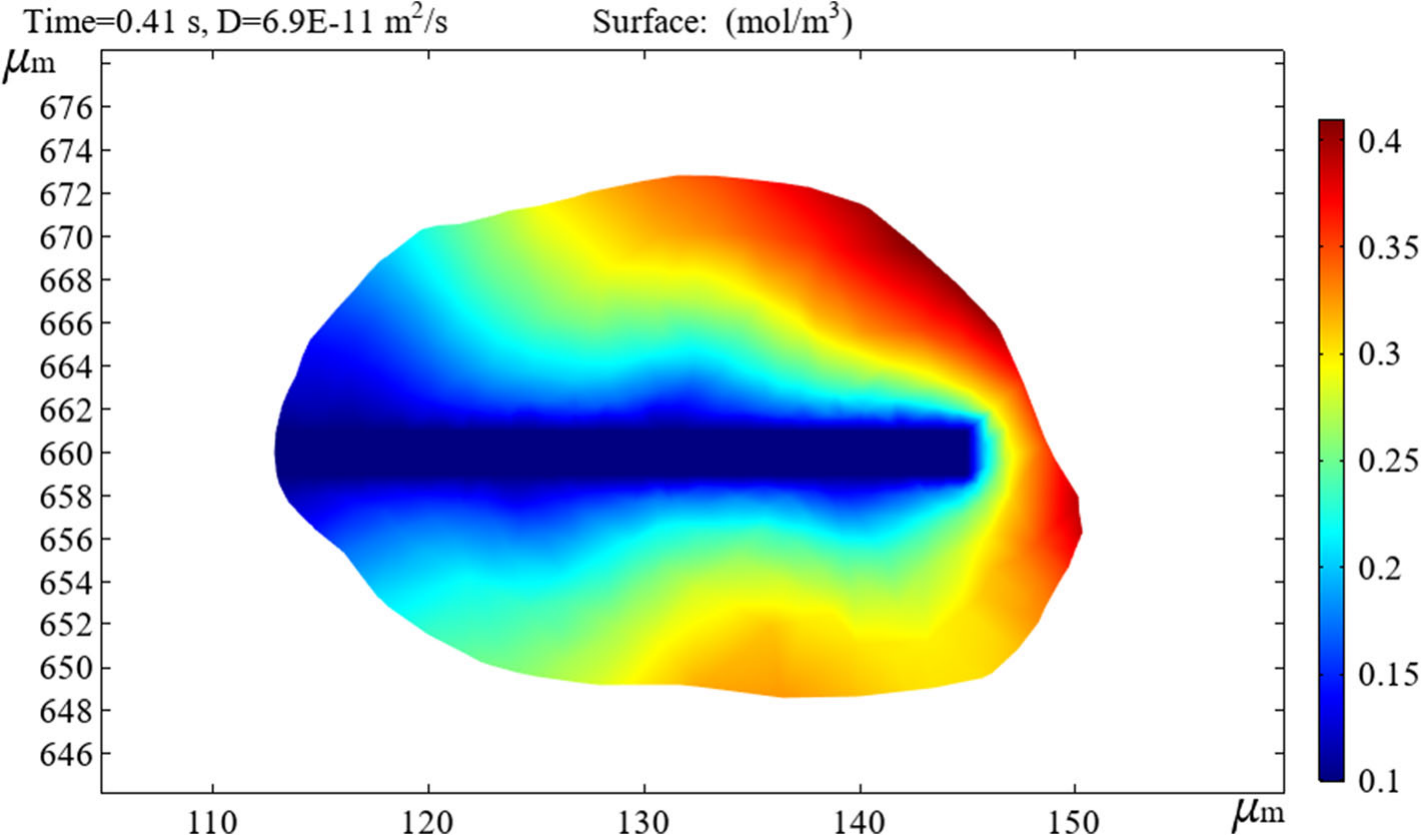
Changes in hematoma surface concentrations

Figure 11 demonstrates that the overall concentration changes on the surface of the hematoma spread along the main tube of the bifurcated tube and gradually spreads outward. For a point on the surface of the hematoma, the smaller the radial distance to the main tube, the more thorough the diffusion.

The analysis above focused on concentration changes on the surface of the hematoma. To study the diffusion and visualization of the hemolytic agent and hematoma, it is necessary to perform two-dimensional cross-sectional analysis inside the hematoma with multiple branch tubes. The drainage tube and hematoma wall outside the hematoma were set to have no flux. We generated a two-dimensional cross section of the overall figure from the symmetry plane of the bifurcated drainage tube. Because there is a concentration gradient between the hemolytic agent and hematoma, after the hemolytic agent enters the hematoma through the bifurcated drainage tube, four times instances were selected randomly. The inter-diffusion dilution reactions observed at each node are illustrated in the figure below.

**Fig. 12:**
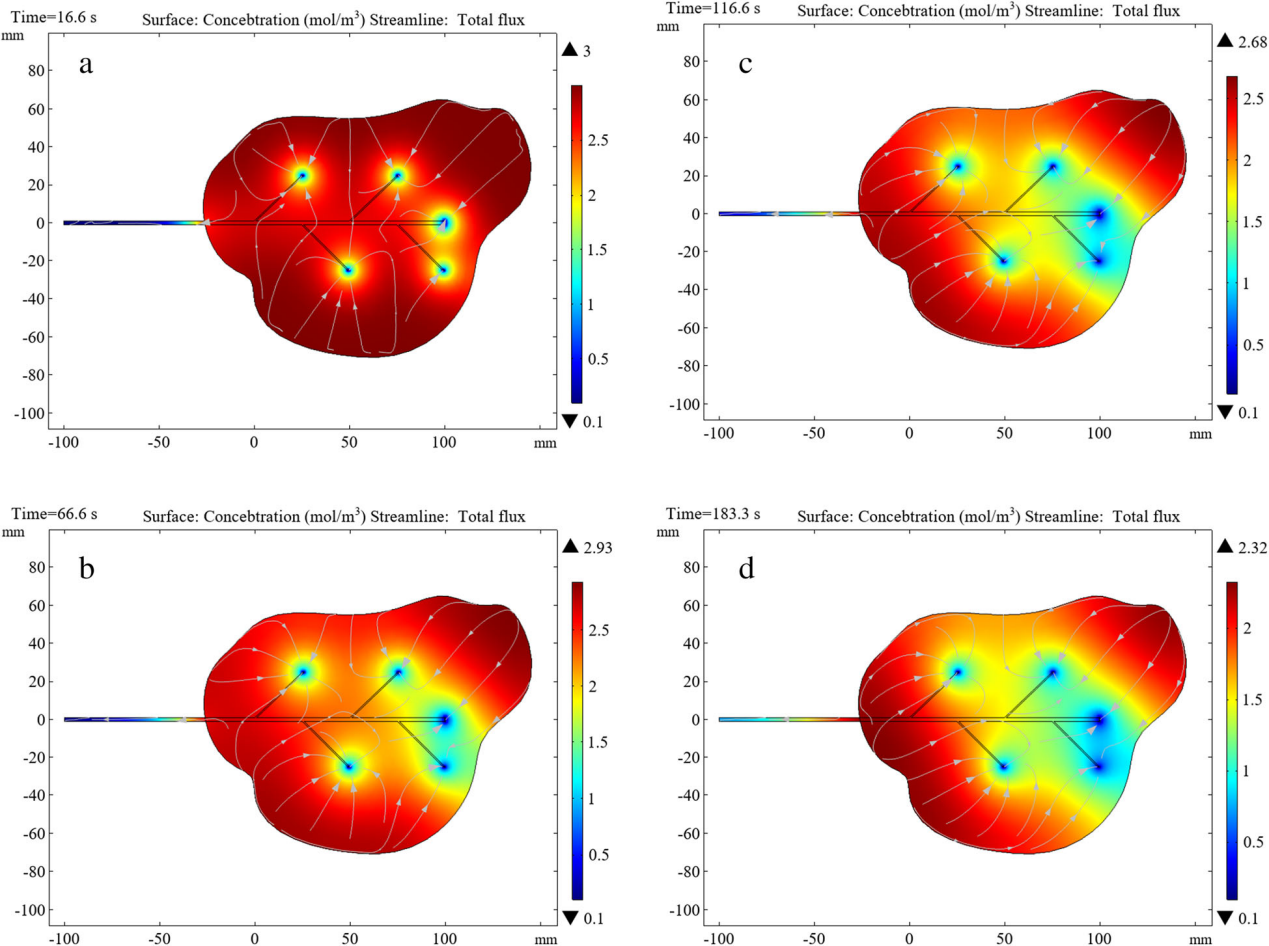
Diffusion concentration and flux changes in the hemolytic agent and hematoma at different times

Figure 12 indicates that the initial hematoma concentration is *c*_1_ = 3 mol/m^3^ and the hemolytic agent concentration is *c*_0_ = 1 mol/m^3^. The colors in the legends in this figure represent changes in the liquid concentration, which are driven by the diffusion process of the hemolytic agent into the hematoma. The lines with white arrows in the figure represent the flux. Changes in direction represent the diffusion process from the hematoma into the hemolytic agent, which is driven by the process of mutual diffusion of binary fluids.

In the case of *t* = 16.6 s, the mutual diffusion reaction is in the initial stages. When the hemolytic agent is drained into the hematoma, the ends of the bifurcated tube begin to change in concentration and flux. In the case of *t* = 66.6 s, with the continuous injection of the hemolytic agent, the agent and hematoma experience the inter-diffusion phenomenon. The hemolytic agent dilutes the concentration of the hematoma and the direction of the hematoma concentration flux points toward the orifices of the drainage tube. When *t* = 116.6 s, one can see that the central part of gradually completes the mutual diffusion reaction with the hemolytic agent. Overall, the hemolytic agent, the part of the hematoma far away from the drainage tube outlets, and the position of the dead corners with complex geometric shapes change more slowly. At *t* = 183.3 s, one can see that the third branch tube, fourth branch tube, and main tube outlet interact more clearly. The hemolytic agent flowing from these three nozzles plays a major role in diffusion. In contrast, the hemolytic agent flowing from the first two branch tubes has a slower diffusion effect in the hematoma.

To make the hemolytic agent spread evenly through the hematoma, it is necessary to improve the design of the bifurcated drainage tube based on this phenomenon. This is one future model application and prospect of this research.

## Conclusions

Based on the surgical removal of intracranial hematomas in clinical medicine, we used the theory of fluid mechanics to study the flow and diffusion of hemolytic agents and hematomas. By using the CFD module of the COMSOL Multiphysics software combined with multiple physics interfaces to perform numerical simulations at different stages of hematoma and hemolytic agent interaction, the design of a bifurcated drainage tube was analyzed and the flow state of the hemolytic agent in the drainage tube and the diffusion of the hemolytic agent into the hematoma were visualized. Both two-dimensional and three-dimensional visual analysis and results were presented.

First, we determined that the flow of the hemolytic agent in the bifurcated drainage tube is dependent on the initial pressure and velocity conditions. Under the initial conditions considered in this article, the agent exhibits a laminar flow state in the main tube and a critical flow state at the bifurcation interfaces. When the required experimental conditions change, the software interface can be adjusted. However, for a specific geometric model, changing the experimental parameters and initial boundary conditions may affect the experimental results. Second, after the hemolytic agent entered the hematoma, the two solutions inter-diffused. This study was conducted while ignoring the chemical reactions of the two agents. However, there are chemical changes in the real world. When considering chemical reactions, visualization software interface settings and simulation results will be more complicated, so more in-depth research is required. Third, when the hemolytic agent entered the hematoma through the drainage tube, the diffusion effect mainly occurred in the exit areas around the last three tubes. According to the visual analysis of this result, we must further improve the design of bifurcated drainage tubes for medical equipment so that they provide the functions of flexible length, multi-angle rotation, import and export, etc. This will be considered in follow-up research as a theoretical basis for the optimal design of medical equipment.

## Data Availability

The datasets used and/or analysed during the current study are not publicly available due to personal privacy but are available from the corresponding author on reasonable request.

## Abbreviation

CFD: Computational fluid dynamics

## References

[1] Rouse H, Ince S (1957). History of hydraulics.

[2] Launius RD (1999). A history of aerodynamics and its impact on flying machines. Technol Cult.

[3] Sun DZ, Lv JP, Waller ST (2011). In-depth analysis of traffic congestion using computational fluid dynamics (CFD) modeling method. J Mod Transport.

[4] Wu X, Xuan YM (2003). Flow and heat transfer model of nanofluids based on lattice-boltzmann method. J Eng Thermophys.

[5] Bothe D, Escher J, Guidotti P, Hieber M, Mucha P, Prüss JW, Shibata Y (2011). On the Maxwell-Stefan approach to multicomponent diffusion. Parabolic problems: the Herbert Amann festschrift.

[6] Sharipov F (2013). Gaseous mixtures in vacuum systems and microfluidics. J Vacuum Sci Technol A.

[7] Chentre N, Saracco P, Dulla S, Ravetto P (2019). On Fick’s law in asymptotic transport theory. Eur Phys J Plus.

[8] Hoshyargar V, Fadaei F, Ashrafizadeh SN (2015). Mass transfer simulation of nanofiltration membranes for electrolyte solutions through generalized Maxwell-Stefan approach. Korean J Chem Eng.

[9] Dzhunushaliev V, Folomeev V, Ramazanov T, Kozhamkulov T (2020). Thermodynamics and statistical physics of quasiparticles within the quark-gluon plasma model. Modern Physics Letters A.

[10] Lehnert W, Meusinger J, Thom F (2000). Modelling of gas transport phenomena in SOFC anodes. J Power Sources.

[11] Li WP (2004). Computational fluid dynamics.

[12] Zou L, Qi Y, Zhao QP (2013). Real-time approach for dynamic liquid simulation using semi-lagrangian. J Softw.

[13] Zhang L, Tang DB, Yang YZ, Yao ZH, Yuan MW (2007). Three-dimensional coupling compact finite difference methods for Navier-stokes equations. Computational mechanics.

[14] Xue Y, Arjomandi M, Kelso R (2011). Visualization of the flow structure in a vortex tube. Exp Thermal Fluid Sci.

[15] Zhang JF, Huang J, Yao W (2020) COMSOL fluid simulation analysis of urban water supply pipeline leakage. China Municipal Eng 000(002):36-41. https://doi.org/CNKI:SUN:ZGSZ.0.2020-02-013.

[16] Fick A (1855). Ueber diffusion. Ann Phys.

[17] Boudin L, Grec B, Salvarani F (2012). A mathematical and numerical analysis of the Maxwell-Stefan diffusion equations. Dis Contin Dyn Syst Ser B.

[18] Zhongfang Technology (2008) Professional numerical analysis system COMSOL multiphysics. CAD/CAM Manuf Informatization (9):40–44. 10.3969/j.issn.1671-8186.2008.09.016

[19] Pan QL, Zhu W, Zhang XL, Chang JC, Cui JZ (2020). Research on a bifurcation location algorithm of a drainage tube based on 3D medical images. Vis Comput Ind Biomed Art.

[20] Chen CX (2016). Stereotactic soft channel minimally invasive hematoma puncture drainage combined with urokinase clot lysis in treatment of hypertensive cerebral hemorrhage. China Med Eng.

[21] Wang YH, Yan HM, Zhang ZX, Wang CD, Wang RH, Wang SX (2006). Experimental and clinical research on urokinase to dissolve and drain intracranial hematoma. Chin J Neurosurg Dis Res.

[22] Yayla S, Ibrahim SS, Olcay AB (2017). Numerical investigation of coalescing plate system to understand the separation of water and oil in water treatment plant of petroleum industry. Eng Appl Comput Fluid Mech.

[23] Sawada S, Itoh T, Misaka T, Obayashi S, Czauderna T, Stephens K (2020). Streamline pair selection for comparative flow field visualization. Vis Comput Ind Biomed Art.

[24] Chen XQ, Jüngel A (2015). Analysis of an incompressible Navier-stokes-Maxwell-Stefan system. Commun Math Phys.

